# “I Believe I Know Better Even than the Psychiatrists What Caused It”: Exploring the Development of Causal Beliefs in People Experiencing Psychosis

**DOI:** 10.1007/s10597-017-0219-3

**Published:** 2018-01-24

**Authors:** Lucy Carter, John Read, Melissa Pyle, Anthony Morrison

**Affiliations:** 1 School of Psychological Sciences, The University of Manchester, Manchester, M13 9PL UK; 2 Clinical Psychology, University of East London, Stratford Campus, 1 Salway Road, Stratford, London, E15 1NF UK; 3 Psychosis Research Unit, Greater Manchester West Mental Health NHS Foundation Trust, Manchester, UK

**Keywords:** Explanatory models, Health beliefs, Schizophrenia, Mental health

## Abstract

This study aimed to describe the causal beliefs of individuals experiencing psychosis, specifically exploring how they are developed and maintained. Individuals with experience of psychosis were recruited from mental health services for in-depth interviews. A thematic analysis was used to analyse transcripts and key themes were identified. Fifteen interviews were conducted. Individuals were engaged in the process of exploring explanations for their experiences and reported sophisticated models of causation. Participants described a change in their beliefs, with the cause of their experiences not immediately clear. Individuals generated their models via external (family, professionals) and internal (evaluative, positive affect) processes and reported differing levels of conviction in relation to their beliefs. Clinicians should take the opportunity to explore the causal beliefs of their service-users, as they are able to provide intelligent and thoughtful explanatory models. In particular, clinicians should be aware of the emotional impact of different aetiological models and their personal role in the development of a client’s beliefs.

## Introduction

When an individual experiences psychological distress they will naturally develop beliefs about these difficulties, which can influence their subsequent behaviour (Baines and Wittkowski 2013). This finding is based on extensive research in physical health, which suggests the importance of exploring a client’s explanatory model as way of facilitating the therapeutic process, as well as to explain variations in human behaviour following a health threat (Kleinman 1978). A number of health belief models attempt to capture how we understand health problems. The most widely used framework, The Self-Regulation Model (SRM) proposes that we conceptualise illness along five dimensions. These include beliefs about consequences, identity/label, timeline (how long the problem will persist) and controllability (SRM; Leventhal et al. 1984). The final dimension, and the focus of this study, is an individual’s beliefs about the cause of their experiences. The development of a causal explanation is considered an automatic process, common to a range of human experiences. We naturally consider why certain events happen and in the case of mental health problems, why they are happening to us (Petrie and Weinman 2006). This process allows us to foresee the consequences of our actions as well as guides our future behaviour and decisions (Lee et al. 1996). Findings have consistently shown that causal reasoning occurs more commonly in situations that are threatening, unexpected and ambiguous; all of which are common to the experience of psychological distress (Kelley and Michela 1980). Numerous theorists have suggested various explanations for this, including; a desire to explain a deviation from the norm, a form of protection against future occurrences and finally to reduce the aversive effect of a distressing event by identifying external and self-irrelevant causes (Weiner 1985).

Studies exploring the causal beliefs of individuals with experience of psychosis indicate that these are complex and varied (Geekie and Read 2009). Often individuals will report holding more than one model (Awan et al. 2015; Jacob 2016; Saravanan et al. 2008) as well as changing their attributions over time (Jacob 2016; Williams and Healy 2001). Overall, service users do appear to prefer a psychosocial explanation of their experiences (Awan et al. 2015; Carter et al. 2016).

How an individual attributes the cause of a health threat has been found to be an important predictor of future behaviour. Current findings indicate that aetiological beliefs can influence, in a logical way, the type of treatment an individual seeks out and the lifestyle changes that they choose to adopt (Weinman et al. 2000). More recently this relationship has been replicated in a small number of studies with individuals experiencing mental health difficulties, particularly in relation to treatment choice and adherence. One study found that individuals with a psychosocial model were more likely to engage efficiently with psychological therapy (Freeman et al. 2013), whilst another study reported that individuals with a biological model reported more favourable opinions of medication (Wiesjahn et al. 2014).

Although current research has explored the personal beliefs of individuals who are accessing mental health services there are very few, if any, that have investigated how an individual’s model is developed and maintained. Models of attribution that aim to understand how we make decisions about the cause of everyday events have identified patterns in the ways in which individuals make these decisions. People will process information using logical (temporal precedence) and selective processes (the most noticeable). Furthermore, a number of models assert that attributions of causality are influenced by the subjective needs of the individual, as well as the objective evidence (Kelley and Michela 1980). They suggest that individuals will develop attributions based on errors, bias and incomplete data gathering, with the aim of promoting a favourable view of the self (Taylor and Brown 1988). For example, people tend to attribute negative events to external factors such as luck, and successful events to more stable traits (Kelley and Michela 1980).

Although patterns in attribution have been identified this has yet to be applied to how individuals conceptualise mental health difficulties. Knowing what factors contribute to an individual’s personal beliefs would provide an insight into the relative importance of different influences as well as the function of specific beliefs. Furthermore, a key component of cognitive approaches to mental health problems, which are now a recommended treatment for individuals experiencing psychosis (NICE 2014), is the development of a shared and meaningful formulation (Carr and McNulty 2014). Understanding what factors may influence an individual’s model of aetiology could possibly aid this process. Therefore, this study aimed to explore, using qualitative techniques, the explanatory models of individuals who have experience of psychosis and what factors contribute to their development and maintenance.

### Methodology

A thematic analysis approach was adopted based on transcripts from semi-structured interviews with individuals who are experiencing psychosis and in contact with mental health services, in the Northwest of the United Kingdom (UK). This method was chosen to allow for a detailed exploration of participant views, without imposing theoretical pre-conceptions or boundaries, in an area with very limited previous research. As with any qualitative approach it is likely that the researcher’s background and personal viewpoints will interact with the research process (Malterud 2001). The lead researcher (LC) works as a research assistant psychologist on trials investigating the effectiveness of different treatment options for people experiencing psychosis, in particular Cognitive Behavioural Therapy (CBT). Therefore, preconceptions relating to this may influence the interview and analysis process. Care was taken to ensure the researcher was reflective in their approach and an independent-rater was asked to identify themes to further guarantee the reliability of the findings.

### Participants

As this study involved human subjects it was subject to an approval from a research ethics committee (NRES Committee North East—Newcastle & North Tyneside 1; 14/NE/1237) and all participants were required to provide written informed consent to participate. A total of 15 participants were recruited from local NHS mental health services across the Greater Manchester area. Participants were required to either; (i) have a diagnosis of schizophrenia, schizo-affective disorder, psychosis or psychotic-like experiences, or (ii) be in contact with an early intervention for psychosis service. They were identified first by their care-coordinator or had expressed previous interest in research via other projects. All participants provided written informed consent.

The majority of the participants were male (10) and were aged between 19 and 57 (M = 33.2). Eight of the participants were in contact with Early Intervention Teams (EIT) and seven were receiving care from Community Mental Health Teams (CMHTs). Clients had been in contact with service on average for 8.2 years. Eight of the participants were categorised as first-episode psychosis (FEP), one had a diagnosis of unspecified psychosis, two had been diagnosed with schizophrenia and four with paranoid schizophrenia. All participants were white British. Interviews lasted on average for 32 min.

### Semi-structured Interview

A semi-structured interview was developed utilising several open-ended questions (with suggested prompts) designed to elicit a participant’s views about; (i) the cause of their experiences and (ii) how these beliefs developed and changed. During the development phase of the interview-guide a service-user reference group was consulted. The interview guide was therefore developed using their advice and feedback. The schedule was always used as a guide throughout the interview process however, the interviewer adopted a curious and flexible approach to allow the participant to fully explore their beliefs without feeling constrained. Interviews ranged between 13 and 52 min in duration and all were digitally recorded and transcribed verbatim.

### Analysis

The research team consisted of a PhD psychology student, who also works full-time as a research assistant on a clinical trial, and three supervisors (two professors of clinical psychology and one post-doctoral researcher) all with extensive experience of working with individuals who have experience of psychosis. Interviews were conducted, recorded, transcribed, coded and initial themes were identified independently by the first-author (LC).

Coding of the data followed the phases of analysis outlined by Braun and Clarke (2006). Within this study it was a realist method that reported the experiences of participants. Once the researcher had familiarised herself with each interview, initial codes for each transcript were produced. This involved working systematically through the entire data set with the aim of identifying features of the data that appeared interesting and relevant to the research question. Initial codes were then collated into candidate themes using an inductive approach. At this stage results were discussed with co-supervisors (TM and MP) and the themes were reviewed and finalised. The reliability of this process was then checked using the third co-supervisor (JR) as an independent-rater. Codebooks listing and describing the themes were constructed by the lead researcher and the independent researcher was asked to code the excerpts using the themes. Two initial attempts resulted in low percentage agreement. Following this, theme descriptions were altered so as to be more concise, and three themes were removed from the analysis as on reflection these were not specific to the research question or lacked sufficient support within the interview data. A final attempt resulted in a higher percentage agreement (69%), which increased to 100% following discussion. This identified three process problems; a theme description that was not adequately detailed (1), a lack of context around the excerpts (3), and independent rater error (4). Finally, a further theme was removed following this discussion as it appeared to be an overall appraisal of the interviews rather than a pattern in the content of the data.

## Results

### Causal Beliefs

All of the participants in this study appeared to welcome the opportunity to talk about their experiences. They provided sophisticated models of causation that were in-depth and personally meaningful. The majority of participants had no difficulty accessing their causal beliefs and even those who were less confident were still able to identify factors that they believed to be relevant. Although participants generally identified a ‘main cause’, the majority considered a number of factors to be relevant in the development of their experiences. Many participants referred to an ‘additive effect’ in which numerous different factors coincided. Some participants articulated that they believed their experiences were real and therefore did not always relate to the term psychosis. However, even these individuals referred to more conventional beliefs (e.g. drug-use, illness-model) during the interview, reflecting on the possibility that there may be an alternative explanation.

It was possible to categorise causal beliefs into four groups; psychosocial; biological; drug use, and unusual beliefs. Psychosocial causes (abuse, bereavement, adulthood trauma) were the most endorsed items by this sample with six people identifying these as their main cause and a further three as a contributory factor. However, the other categories of beliefs were also frequently referred to throughout the transcripts reflecting the multi-factorial nature of an individual’s conceptualisation. This included a total of six individuals referring to biological or genetic attributions (two main), eight referring to unusual beliefs (two main) and finally six participants viewing drug-use as a possible contributory factor (one main). The findings from the thematic analysis are presented below, illustrated by quotes from the transcript, which aim to reflect the participant’s understanding.

### Development and Maintenance of Causal Beliefs

#### Moving from Believing Experiences Are Real Perceptions to Needing a Causal Explanation

Nine interviewees referred back to a point in time in which they didn’t realise that their experiences were unusual and report a change in their beliefs from the onset of their experiences to now. At the beginning there is no requirement for a causal model as they interpret their problems as a ‘normal’ or real-life experience. This includes participants discussing a previously held belief that hearing voices was a normal occurrence, which eventually changes;

“I never really thought too much into it, I always assumed it was normal being at a young age hearing voices it was only until I was getting older that I was you know, when I was saying to my mates you know, I seen the expression on their faces like, like wow something is not right.” (PP11)

Whilst others refer to strongly held beliefs that have now altered;

“Yea, I just accepted it was life, I thought I was being very post-modern and going beyond reason. I was poorly but I didn’t know what was wrong, like I said I was accepting it as part of my life now and then he [psychiatrist] kind of took me to one side and he said [name] you have got schizophrenia, so I went, okay.” (PP14)

#### Experiencing Negative Affect Associated with Psychiatric Diagnosis

Many interviewees (10) described an adverse response to being given a casual explanation for their experiences which involves a psychiatric diagnosis or label (e.g. psychosis, schizophrenia). This included feelings of shame, guilt and confusion;

“Then he [psychiatrist] kind of took me to one side and he said [name] you have got schizophrenia, so I went, okay. I felt guilty if I am honest, I felt I have been doing things wrong”. (PP14)

Furthermore, four participants expressed concern about how this label may be perceived by others;

“Once it kind of hit home that I have got psychosis I need to get help I was a bit gutted because it’s not a nice thing to have or to tell people that you have had as well so yes I was a bit worried about that.” (PP10)

#### The Cause Is Not Immediately Obvious

Participants reported that the aetiology of their experiences was not immediately clear. A total of six interviewees discussed a difficulty in understanding how their experiences had developed. Some participants described a process in which they had, over-time, made sense of their experiences and formulated a causal model. However, there is a clear indication that this was not an immediate process;

“So it has taken a while for me to come to them sort of conclusions over a period of time, penny’s dropping.” (PP3)

Other’s reported still feeling unsure about the cause of their experiences, but with a sense that they would like to understand why;

“There has got to be a reason why, that’s what I wanted to find out, if you get a cut on your hand you can see it, you can see it getting worse, whereas something inside you can’t see. There is nothing obvious.” (PP7)

#### Evaluate Psychosocial Causes and Make a Decision About Their Relevance

Nine participants considered or searched for environmental causes and subsequently evaluated whether or not they thought that events in their lives had caused their experiences. This included general statements that referred to an individual searching for an environmental trigger; “I have no idea…..I was never abused or anything there’s nothing like that” (PP1). Whilst others reflected on specific life events and evaluated their relative importance. For example, one participant appeared to resolve that a previous trauma was the trigger to her difficulties;

“I think it might have something to do because I was sexually abused when I was fourteen. I think because that’s the one that caused the most trauma to myself, because after that I just went completely off the rails.” (PP12)

Conversely, another participant concluded that his previous life circumstances were not associated with his current difficulties;

“I can’t see being locked away in bedrooms and cupboards and abused and being in the care system is the cause of it because I don’t believe it in myself” (PP11)

#### Understand Experiences Based on Their Pre-conceptions of Psychosis

Some participants conceptualised their own experiences based on their pre-existing ideas about the cause of psychosis and the ‘type’ of person who would usually develop these difficulties. Five interviewees referred to an understanding of their experiences which was partially based on their previous knowledge/experience of mental health problems (e.g. via work, study, family members).

“When I were 15 apparently them workers turned to my mum and said he’s a psychopath, you know me step-father, he’s never going to change…. you know but I do worry I’m a psychopath.” (PP2)

For some participants (3) this involved rejecting the association with this ‘group’ as they were unable to reconcile the similarities between this ‘group’ and themselves;

“So when I had homeless patients coming in to me …….in all fairness, they drink a lot ……I can understand why they might be seeing things more. Whereas I don’t really drink, I don’t do drugs, so its kind, kind of two different, it is two different backgrounds.” (PP7)

#### Attribute to Factors That Have a Positive Impact on How They Feel

A high number of interviewees (10) conceptualised their difficulties in a way that appeared to increase their positive affect. Furthermore, participants described different emotional reactions to the various models of causation. For some this involved attributing their experiences (or an aspect of them) to spiritual origins and expressing a positive emotional reaction to this belief:

“I think I was touched by the Holy Spirit, that felt good, like I was being good or being rewarded for being good, whether that’s to do with schizophrenia I don’t know, it’s a good feeling, it is positive.” (PP14)

For others it involved associating their experiences with positive characteristics about themselves e.g. over-concern about others or having an intellectual mind; “I always make sure other people are happy rather than myself and it has obviously not worked.” (PP7). Another participant externalised the cause of his difficulties, which he felt reduced the blame attributed to himself;

“It was maybe making the most of the weaknesses, that perhaps, the small weaknesses that I did have, that probably wouldn’t have shown if it hadn’t have been for those sort of things….I suppose it gives me some sort of comfort that it’s almost like external circumstances.” (PP3)

#### Reluctance to Attribute Cause to Drug-Use

Many interviewees referred to substance misuse when considering possible beliefs about the cause of their experiences, however amongst these individuals there was a general reluctance to accept this as cause. Of the six participants who discussed drug-use as part of the aetiological discussion, five of these either; (i) Accepted that narcotics may have potentially played a part alongside other contributory factors, but did not view it as a solitary cause;

“Ermm, I think it was definitely a contributing factor but I don’t think it was the sole reason why I lost my marbles.” (PP10)

Or, (ii) considered the relative influence of drug-use on their experiences and concluded that this had not contributed:

“I still smoke cannabis, I have to do though for anger issues….I have always looked at my illness and my drug habit and gone you know, as one caused the other, and it’s not, it’s not like that…..what is the point in taking something I enjoy away doing, I enjoy it.” (PP15)

#### Discuss with Others and Evaluate Their Opinions

Discussing the cause of their experiences with others, and subsequently evaluating the beliefs held by others, was evident across 11 of the transcripts. In total there were thirteen examples of this process (two participants reported discussions with more than person). It appeared that individuals sought out the opinions of others, but differed to the degree in which they endorsed what others suggested as possible contributors. Some reported accepting the suggestions provided by others as possible (8); “[care coordinator] said that is a possibility (bereavement) and when you think about it, it is true.” (PP7).

Whilst, other participants reported only a partial agreement (3);

“I mean I have spoken to people about it and they tend to think it was all the cannabis, they think that it was just smoking weed, but when I look back I see, I see it as that and also, well I see it as three things.” (PP10)

Finally, two participants reported disagreeing with the models provided by other people as it doesn’t appear realistic according to their own beliefs;

**Interviewer**: Then you said that you don’t feel like that it was, you can’t see how it [bereavement] is a cause, why is that, why do you think that that?

**Participant**: “I just think it was a coincidence at the time, that’s all you know, it was just one of them things, because death and that doesn’t faze me, doesn’t bother me in the slightest.” (PP15)

#### Professionals Do Not Offer a Causal Model

When explicitly asked about the provision of a causal model by professionals, the majority of the interviewees (9) reported having not received this information from members of their care team;

“Although I have had experience with a psychiatrist, none have really elucidated that much really….nobody has come up with it probably comes from there.” (PP3)

Some participants referred to being given a psychiatric diagnosis as an explanation however, a specific cause was not provided;

“Not really, it was just a quick to the point conversation, you have got schizophrenia, you okay, yes okay and he was off to the next person.” (PP14)

#### Differing Conviction in Beliefs

When discussing their causal models it is evident that whilst some were confident about their beliefs, others felt less sure and were more open to alternative explanations. A number of participants (4) expressed a fixed model with a clear understanding of how their experiences developed;

“I’m pretty sure now that’s where my problems arise, do you know what I mean. I mean I’ve come to them conclusions…..Yea I mean I’ve got a fair idea where these sort of symptoms, where my problems have arisen.” (PP3)

Others reported feeling unsure about the cause and deliberated over various different explanations during the discussion (3). This tended to be those who had only recently accessed services or those that expressed less interest in the cause of their problems;

“I have been asked this question before (why did it happen) and I can’t actually come up with a reason why, I don’t know, I just don’t get it.” (PP6)

#### Awareness of a Discrepancy Between Contradictory Beliefs

Some interviewees reported understanding their experiences using different models that are in conflict with each other. A total of six participants reported still holding an unusual aspect to their beliefs alongside a more conventional model, and there was a recognition that these beliefs cannot be held in conjunction;

“Yea i can look at it from both ways…..psychosis and my way, I can look at it from both sides and I know how ridiculous it sounds….this is the catch 22.” (PP1)

When discussing their beliefs, participants articulated they are able to understand their experiences using both models, and that their opinions are changeable;

“I just thought that like my neighbours were listening to conversations that me and my husband were having….I still to this day, I still think something happened which is probably why I’m still on me medication but I’m not 100% sure that it happened.” (PP5)

#### Disinterest in an Aetiological Model

A few interviewees (4) expressed disinterest in relation to the cause of their experiences. When asked about how much they thought about the aetiology of their experiences or how important this was, they reported not engaging with the development of a model because of a reluctance to dwell on their experiences. Furthermore, there is a view that understanding the cause will not improve their situation or is a pointless process;

“I have not really put thought into it because it’ll just mess my head up if I put thought into it….I don’t want to mess around with something like because it’s just pointless thinking about it, there’s nothing it’s just fruitless.” (PP15)

“Erm not really, I have kind of just been getting on with it because I know its something that I am probably going to have for the rest of my life….I thought I just need to get on with it because sitting and dwelling about it is not going to do me any good.” (PP6)

## Discussion

This study elucidated participant’s beliefs about the cause of their psychosis. Overall, individuals appeared to evaluate possible causes using both internal and external processes. Generally, there was a preference for a psychosocial explanation of their experiences, however many factors were considered to be of importance. In line with previous research (Geekie 2013), most participants placed great value in understanding the cause of their experiences. For many, a ‘search for meaning’ appeared to be an automatic and active process following the onset of their difficulties. Furthermore, no two individuals reported identical models, reflecting the complex multifactorial pathway to the development of psychosis.

### Initial Development

The majority of participants did not initially understand their experiences (e.g. hallucinations and beliefs) as a mental health problem. Instead they were either interpreted as real perceptions (e.g. felt their beliefs/paranoia was grounded in reality) or as normal/common experiences (e.g. believing everyone hears voices). It is likely that as the distress associated with their experiences increased the individual sought help from mental health services and an alternative explanation was provided. Indeed, previous research suggests that it is the distress associated with unusual experiences that distinguishes clinical from non-clinical samples (Peters et al. 2016). Furthermore, one study reported that individuals experiencing their first-episode of psychosis did not attribute their experiences to psychosis until they came into contact with services (Judge et al. 2008).

### Positive and Negative Affect

There was a tendency for some individuals to attribute their experiences to causes that contributed to a positive self-conceptualisation. This pattern is reported throughout the attribution literature. This has been explained as a pre-disposed human bias in which individuals are naturally more attentive to positive compared to negative aspects of themselves (Taylor and Brown 1988). At a time when many individuals are often experiencing high levels of distress, promoting a positive self-image would possibly reflect a form of self-protection. Similarly, individuals reported negative affect when provided with an explanatory model that included a psychiatric label or diagnosis, as well as a reluctance to be associated with individuals who would usually have these experiences. Research has found that individuals adopt alternative causal beliefs (to the medical model) in an attempt to avoid the stigmatizing association with ‘mental illness’ (Saravanan et al. 2008). Indeed, one recent study of psychology students demonstrated that diagnostic labelling can increase perceptions of dangerousness and unpredictability towards people experiencing psychosis (Magliano et al. 2016). It is therefore reasonable that individuals will experience negative feelings towards this label, as well a reluctance to share this information with others. Within western culture, individuals experiencing the ‘symptoms’ of psychosis are often encouraged to view them as this, as opposed to a spiritual/alternative interpretation. It could be that individuals would benefit from being free to retain these beliefs if they have a positive influence on their emotional well-being.

### Information Processing

Research has found that people will associate two events in a causal manner if the events co-vary (one is present/absent in line with the second event), occur in close proximity to each other and the cause precedes the event. In relation to drug-use and psychosocial factors these processes appeared to be employed to evaluate the relevance of these models to an individual’s own experiences. Participants tended to reject the concept that substances had caused their experiences alone because the two events did not always occur together and drug-use did not always precede their distress. Some individuals reported that narcotics helped with the management of their experiences, indicating that within their conceptualisation it was an outcome of their difficulties as opposed to a trigger. Indeed some researchers suggests that many individuals use drugs to alleviate other symptoms typical of individuals with a diagnosis of schizophrenia such as anxiety and depression (Asher and Gask 2010; Hambrecht and Häfner 2000). Similarly, participants in this study did not attribute their experiences to adverse childhood circumstances as those with environmental models tended to identify factors that occurred in adolescence or adulthood and in close proximity to the start of their experiences.

Empirical evidence also suggests that individuals will use previously held beliefs about an event when evaluating causal factors. Many participants in this study based their understanding of their experiences on their own pre-conceptions of psychosis. Indeed, people reported searching for extremely significant adverse events when attempting to understand their experiences, with a sense that what could be considered less minor life events (e.g. bullying, bereavement) were not enough to explain their level of distress.

### Social and Cultural Influences

All of the participants in this study were white British, and articulated beliefs that would be considered culturally acceptable in the developed world. Research has consistently demonstrated that ‘illness beliefs’ are almost always culturally shaped (Kleinman 1988). More directly, participants referred to models of aetiology provided by family, friends and professionals. Individuals appeared to process these opinions according to the requirements discussed above and therefore did not always share agreement. This finding suggests that individuals seek out other people’s opinions; however, they retain a very personal model that fits with their understanding. The finding that professionals did not offer a causal model is supported by previous research, which found that only a low number of consultants reported ‘always’ offering an aetiological model (Bhui and Bhugra 2002). However, it appeared that a formulation of how an individual’s experiences developed was discussed in a less formal or explicit way as part of the therapeutic process for some of the interviewees. Finally, a number of participants’ family members attributed cause to drug-use. This is perhaps an attempt of care-givers protecting themselves from the possible development of these experiences. Some findings have suggested that ‘observers’ will make internal attributions (place blame on the individual/their actions) as a way of maintaining a ‘just world view’ (Kelley and Michela 1980).

### Disinterest in Aetiology

A number of the participants expressed disinterest in understanding the cause of their problems. This dichotomy could potentially be understood using the two recovery styles that are typically observed in people experiencing mental distress. Those who are considered to be ‘integrative’ are flexible in their thinking and incorporate their experiences into a positive aspect about themselves. Conversely those who ‘seal over’, isolate the distressed part of themselves from other aspects of their lives and view it as an annoyance (McGlashan 1987). Differences have been identified in long-term outcomes between the two recovery approaches, in particular quality of life and overall functioning (Thompson et al. 2003). Whilst the majority of individuals in this study expressed interest in their causal models there are number of participants who express disinterest, possibly reflective of a sealing-over recovery style.

### Limitations

This study has the following limitations. Firstly, the study group of participants who have experience of psychosis represents a very small number of this group in total and may be biased by the self-selecting recruitment approach. Those who agreed to take part may have more interest in reflecting on their causal beliefs, as well as being more willing to talk about their experiences. Therefore, they may not be generalizable to the population as a whole. Finally, as noted above, this study interviewed participants of one ethnicity and there are documented differences in the causal beliefs of different ethnic groups (McCabe and Priebe 2004).

### Future Research

Future research should focus on exploring possible relationships between different causal beliefs and their emotional/behavioural impact. It would also be interesting to look qualitatively at cultural specific beliefs, particularly as they have been associated with different outcomes in previous research. Furthermore, additional research looking at the relationship between recovery styles and the attribution process would be interesting, particularly as an indication of future outcome. Finally, research investigating how or whether clinicians elicit the personal beliefs of the individual as part of the therapeutic process would also be worthy of future research. Particularly as this is a key recommendation from a recent independent review on how to improve care for individuals experiencing psychosis (The Schizophrenia Commission 2012).

### Clinical Implications

The causal beliefs of individuals with psychosis should be explored as part of the therapeutic process to allow individuals the opportunity to reflect on their experiences. It is evident that many people value the opportunity to discuss what may have caused their experiences and hold a lot of insight into why their experiences may have developed. Clinicians should be aware of the potential positive aspects to holding different beliefs and that many people hold pre-conceptions about psychiatric diagnoses that may need to be explored. Furthermore, people do not appear to naturally associate events that do not occur in close proximity and therefore childhood experiences may need to be more explicitly discussed and linked with an individual’s current difficulties. For those individuals with more unusual beliefs, clinicians need to allow the client the opportunity to express these opinions without judgment, particularly as the research suggests that holding alternative interpretations (e.g. spiritual beliefs) can reduce the distress associated with psychotic experiences. Finally, external influences are also an integral part of the belief development process and clinicians hold an important role in how an individual understands their experiences, particularly because of the ambiguity that many people can feel when their experiences initially develop.
